# Lymphoma versus Carcinoma and Other Collaborations

**DOI:** 10.3390/cells11010174

**Published:** 2022-01-05

**Authors:** Karen Pulford

**Affiliations:** Nuffield Division of Clinical Laboratory Sciences, Radcliffe Department of Medicine, University of Oxford, Oxford OX3 9DU, UK; karen.pulford@ndcls.ox.ac.uk

**Keywords:** immunohistochemistry, lymphoma diagnosis, monoclonal antibodies, cancer biomarkers, CD antigens, ALK, anaplastic large cell lymphoma

## Abstract

David Mason started his research career at a time when lymphoma diagnosis was based primarily on cellular morphology, clinical symptoms and special cytochemical stains using formalin fixed tissue sections. There were occasions, however, where the morphology was unhelpful, such as in the case of anaplastic or poorly differentiated tumours, where a distinction between lymphoma and a non-haematopoietic tumour was often problematical. Accurate diagnosis became even more important with the developments in the clinical staging of lymphoma and the availability of more effective treatments. One way forward to improve diagnosis was to use immunohistochemistry to study the antigens expressed by the tumor cells.

## 1. Background

Immunohistochemistry has come a long way since the immunofluorescence technique used by Coons et al. [1]. Later immunoenzymatic labelling techniques using horseradish peroxidase (HRP)-conjugated primary [2] and secondary antibodies [3] permitted the sites of antigen/antibody binding to be visualised using light microscopy. This opened up opportunities for immunohistochemistry to be used within a clinical diagnostic context [4,5]. This was the ‘state of play’ when David Mason started his research career in haematopathology.

## 2. First Steps

David Mason laid the foundations for his future research by collaborating with Professor Clive Taylor [6]. Using HRP labelled polyclonal antibodies, they reported the presence of intracellular immunoglobulin light chain in formalin-fixed sections of multiple myeloma. The technique was reproducible, sensitive and, importantly, allowed the study of archived biopsy material. David then went on to confirm the use of immunoperoxidase for detecting proteins such as lyzoyzme and lactoferrin in myeloproliferative disorders [7]. Not content with using just one color, David Mason and Rita Sammons developed and explored the use of alkaline phosphatase as an alternative immunoenzymatic label for studying formalin fixed tissues and cell smears leading to the option of double labelling of cells [8].

All this was useful but diagnostic immunohistochemistry was still hindered by the limited number of antigens that could be studied, the sensitivity of the antibodies to routine histological fixatives as well as the specificity of the antibodies. The lack of available reagents and the problem of specificity were key objectives to be overcome in the next phase of David’s research career. Monoclonal antibody technology and developments in antigen retrieval techniques came to the rescue for both David Mason and his colleague Kevin Gatter who started the Cancer Research Campaign, later the Cancer Research–UK (CR-UK) Group.

## 3. Launch of the LRF Immunodiagnostics Unit

David Mason saw the advantages of using monoclonal antibodies to increase the number of antigens that could be studied and improve the specificity and reproducibility of immunolabelling. He recognised their value for improving diagnosis and classifying tumours. With funding support from the Leukaemia Research Fund (LRF, now known as Blood Cancer UK) David established the LRF Immunodiagnostics Group. It was an exciting time of trial and error and the world of the Cluster of Differentiation (CD) classification of antigens was at least two years away. The aim of the Group was twofold; (a) to produce and use monoclonal antibodies recognising white cell antigens and (b) produce antibodies that recognised formalin resistant epitopes. The overriding principle of its work was encapsulated by the quote from one of David’s and Clive Taylor’s papers that “the accuracy of the technique can never be greater than the specificity of the anti-sera used” [6].

All the antibodies produced by the LRF Immunodiagnostics Group were fully characterized using immunolabelling, biochemistry and flow cytometry initially and, when the technologies became available, through the use of transfectants and functional studies before being ‘let loose’ on the research communities. As the Group became more established it was promoted to the LRF Immunodiagnostics Unit in recognition of its work. It is notable that the charity Blood Cancer UK (through all its changes in title) provided continued funding for the Unit for more than 29 years in the form of Programme and Project Grants thus showing their confidence in its research.

## 4. Overcoming the Fixation Problem—Lymphoma or Carcinoma?

I joined Jacqueline Cordell and Rita-Elizabeth Woolston in 1981 after finishing a PhD in London. A 1982 Oxford study had confirmed the value of using antibodies directed against different cell types to distinguish between lymphoma and carcinoma, and importantly, to establish a diagnosis when conventional methods had been inconclusive. Further work using this panel was, however, hampered by the fact that most of the antibodies did not work in routinely fixed tissues [9]. One of my early tasks was to produce and characterise an antibody that recognized a leucocyte-specific antigen in routinely fixed tissues. A density gradient separated suspension of lymphoma cells from a patient with a T-cell lymphoma was used as an antigen source. Resulting hybridomas were screened on cryostat sections of tonsil, a rapid screening test that enabled the reactivity of any antibodies to be seen on a wide range of cell types. Excitingly, one antibody stained only haematopoietic cells but not epithelium or endothelium.

**Figure 1:**
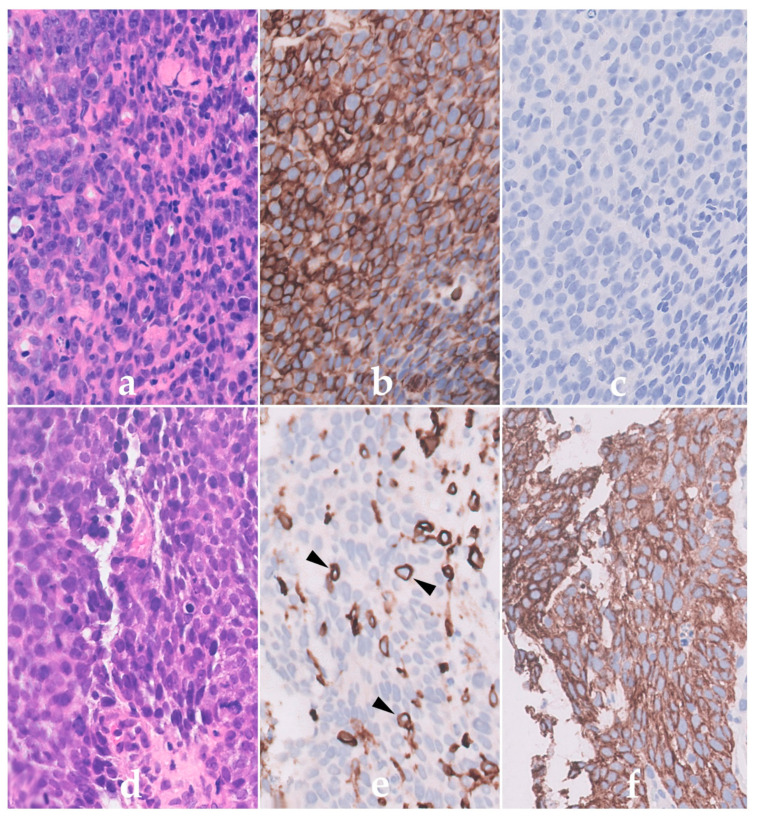
Immunoperoxidase labelling of formalin sections of a Non-Hodgkin’s lymphoma lymphoma (NHL) (**a**–**c**) and a carcinoma (**d**–**f**) using the anti-CD45 antibody PD7/26. Haematoxylin and eosin labelling shows the presence of cells with a similar morphology in (**a**) the NHL and (**d**) carcinoma. The NHL cells in (**b**) are however strongly positive for CD45 (brown) but lack cytokeratin (**c**) confirming their haematopoietic origin. In contrast, the epithelial cells of the carcinoma are (**e**) CD45 negative but (**f**) express cytokeratin. Note the labelling of the scattered normal CD45-positive lymphoid cells (arrowed) within the carcinoma in (**e**).

A quick test on trypsin treated paraffin sections of tonsil confirmed the specific labelling staining only of haematopoietic cells and, importantly, that the antibody recognized a formalin resistant epitope. This monoclonal antibody, 2B11, was later joined by another antibody PD7/26 produced by Jacqueline Cordell. This reagent exhibited a slightly different staining pattern but also recognised a formalin resistant epitope on leucocytes. Western blotting techniques introduced into the Unit by Kingsley Micklem (joining in 1986) later showed both antibodies to label high molecular weight 200 kD proteins. Antibodies 2B11 and PD7/26 were assigned to be anti-CD45 and CD45RB, respectively, at the second Human Leucocyte Differentiation (HLDA) Workshops held in Boston USA. These two antibodies were used in a key international study of human normal, hyperplastic, neoplastic lymphoid tissues as well as non-lymphoid neoplasms all of which had had undergone routine histological fixation [10]. Results confirmed the value of using these leucocyte specific antibodies for aiding lymphoma diagnosis. An example of labelling is shown in Figure 1.

The importance and effectiveness of a combination of these two antibodies and immunohistochemical labelling for the diagnosis of poorly differentiated or anaplastic tumours were highlighted in two later papers from Oxford [11,12]. A further important aspect of these papers was the addition of follow-up clinical details. This step was important since a diagnosis of lymphoma was associated with different treatments to that for carcinomas. The paper concluded that there was an urgent need for the accurate diagnosis of lymphoma because of treatment and prognosis. It also indicated that there was probable under reporting of lymphoma. The recommendation was made that since the techniques in immunohistochemistry were relatively simple and could be used on routinely processed tissue, immunohistochemistry should be carried out more widely in diagnostic histopathology laboratories. Subsequent advances in the pretreatment of routinely fixed biopsy sections, such as heat- and pressure-based treatments and the use of different buffers, has enabled many more antigens to be revealed thus increasing the numbers of diagnostic markers that can be studied.

David Mason and Kevin Gatter confirmed the advantages of using a panel of antibodies for diagnosis [9,11]. The next logical step for both the LRF Immunodiagnostics Unit and the Cancer Research–UK (CR-UK) Group, now containing Helen Turley (joining in 1984) was to produce monoclonal antibodies to antigens expressed by different types of leucocytes to improve diagnosis and in doing so the classification of haematological malignancies. The LRF Unit was successful in producing more than 80 monoclonal antibodies recognizing different cell types during the next 30 years, of which 43 recognized their target antigen in routinely processed tissue biopsies. Many of these were specific for CD antigens, for example, CD3, CD4, CD8, CD10, CD15, CD16, CD20, CD68, CD79a, CD45, CD45RA, CD45RB, CD66 and CD246 while others recognised intracellular molecules such as BCL-2 and nucleophosmin. More than 15 monoclonal antibodies made by David and the LRF Immunodiagnostics Unit are currently used in routine diagnostic panels throughout the world.

## 5. Collaborations and Classifications

The skills and enthusiasm of David Mason as an avid networker and multilinguist coupled with the availability of well characterized antibodies led to multiple national and international collaborations. David was greatly assisted in this by the organizational and administrative skills of Bridget Watson who joined his group in 1985. He established early long-lasting collaborations with haematopathologists such as Professor Brunangelo Falini, Perugia, Prof Harald Stein, Berlin, Prof Georges Delsol, Toulouse, Prof Roger Warnke, Stanford and Prof Teresa Marafioti (later becoming his wife) to name but a few. These links provided a platform for David to broaden his research. Not only did the LRF Immunodiagnostics Unit receive many varieties of antigens (cells, peptides, recombinant proteins) from multiple sources but, as the number of antibodies produced within the group multiplied, so did the opportunities to send reagents to other researchers. David Mason (and Kevin Gatter) frequently came to the laboratory before leaving to attend a meeting to pick up handfuls of samples of antibodies to give away.

David was very welcoming to researchers and the Oxford laboratory became a haven for visitors from as far away as Australia, New Zealand, USA and Malaysia. Indeed, people from all five continents have visited Oxford and worked alongside members of the Unit to take the techniques back home. This was a two-way street and the Unit benefitted greatly from friendships made and repeat visit invitations. David Mason also supervised many DPhil. students who went on to become leaders in their fields, examples being Prof Paul Moss (Prof Haematology and Head of the School of Cancer Sciences and leading the UK Coronavirus Immunology Consortium, Birmingham) and Prof Wendy Erber (Chair and Head of the School of Pathology and Laboratory Medicine, Perth).

David Mason championed the HLDA Workshops. Starting in 1982, these Workshops were held to obtain some order in the nomenclature of monoclonal antibodies. Rather than being known by their laboratory names, antibodies were identified by their target antigens and the term CD was proposed. David saw the importance of this effort and participated in all these global collaborative events, forming part of some organizing committees and acting as Chair of the Harrogate meeting in 2002. Although the Unit was heavily involved in all of these meetings, Margaret Jones (joining in 1985) played a major role in studying and reporting on the majority of the antibodies submitted to the B-cell panels.

Efforts to spread and gather information by David Mason and Kevin Gatter were crucial to advances in the classification of lymphomas. Both David and Kevin were two of the founder members of the International Lymphoma Study Group (ILSG). This group consisted of nineteen haematopathologists from all over the world, many of whom had visited Oxford to work with David and Kevin. The ILSG worked together to introduce the Revised European-American Classification of Lymphoid Neoplasms (R.E.A.L.) in 1994 [13]. This system was the first to define diseases based on the immunophenotype as well as the morphological, clinical and genetic properties of the neoplastic cells. It gained widespread use and was revised and was updated to include information from gene expression profiling and was integrated into the World Health Organisation of Tumours of Haematopoietic and Lymphoid Tissues published in 2001 [14].

It seems appropriate here to describe one example of bringing together a long-standing research interest of David Mason with immunohistochemistry, the strengths of the LRF Immunodiagnostics Unit and collaborators. David Mason had, for many years, been fascinated by anaplastic large cell lymphoma (ALCL). As its name suggests, these tumours exhibited great heterogeneity in their morphology, clinical features and immunophenotype and were extremely difficult to diagnose. David was one of the researchers who reported the expression of CD30 by ALCL leading to the description of ALCL as Ki-1 lymphoma [15]. CD30 was not, however, specific for ALCL. In 1985 David and co-workers described that the t(2;5) translocation was associated with ALCL [16] and in 1994 the resulting fusion protein nucleophosmin–anaplastic lymphoma kinase (NPM-ALK) was sequenced by two different groups in Memphis, USA [17] and Tokyo, Japan [18]. David contacted Prof Steve Morris and the Oxford-Memphis collaboration started.

Alison Banham (joining in 1994) produced ALK recombinant protein which was used to produce the ALK1 antibody. Use of this antibody demonstrated NPM-ALK protein to be present at high levels in ALCL resulting in the identification the new tumour entity ALK-positive ALCL [19]. This lymphoma, ALK-negative ALCL and ALK-positive large B-cell lymphoma were formally recognized in the 2008 WHO classification [20]. This not only provided important diagnostic and prognostic information since ALK-positive lymphomas have an improved outlook, but an important offshoot of this work was that the use of this antibody has opened up pathways to investigate tumor development and therapy. An interesting twist of fate is that work by David and the Unit on ALCL has led to the world-wide investigation of ALK protein in both solid as well as haematological tumours by other workers.

## 6. Conclusions

David Mason was one of the pioneers in immunohistochemistry with the vision of linking immunohistochemistry to pathology as a way of improving diagnosis, a vital step for the appropriate treatment of patients. His legacy, and that of Kevin Gatter, of producing and using monoclonal reagents as diagnostic tools has helped change the practice of diagnostic pathology worldwide. Their work has not only played key roles in the improved identification of haematopoietic tumours but has provided reagents to understand tumour development and the quest for improved treatment options.

David led the LRF Immunodiagnostics Unit for more than 29 years. A remarkable feature of the Unit was the length of time that its core members stayed with the group (totaling more than 132 years). The prolonged existence of the Unit with its core members was often remarked upon and was a testament to the quality of David’s leadership, his research aims and abilities to inspire as well as the respect and friendship within the Unit and its collaborators.

## Data Availability

Not applicable.
